# Culprits of PDAC resistance to gemcitabine and immune checkpoint inhibitor: Tumour microenvironment components

**DOI:** 10.3389/fmolb.2022.1020888

**Published:** 2022-10-10

**Authors:** Sheng-Kai Hsu, Mahendra Jadhao, Wei-Ting Liao, Wen-Tsan Chang, Chun-Tzu Hung, Chien-Chih Chiu

**Affiliations:** ^1^ Department of Biotechnology, Kaohsiung Medical University, Kaohsiung, Taiwan; ^2^ Department of Medical Laboratory Science and Biotechnology, Kaohsiung Medical University, Kaohsiung, Taiwan; ^3^ Department of Medicinal and Applied Chemistry, Kaohsiung Medical University, Kaohsiung, Taiwan; ^4^ Department of Cancer Biology, University of Cincinnati College of Medicine, Cincinnati, OH, United States; ^5^ Division of General and Digestive Surgery, Department of Surgery, Kaohsiung Medical University Hospital, Kaohsiung, Taiwan; ^6^ Department of Surgery, School of Medicine, College of Medicine, Kaohsiung Medical University, Kaohsiung, Taiwan; ^7^ Center for Cancer Research, Kaohsiung Medical University Hospital, Kaohsiung Medical University, Kaohsiung, Taiwan; ^8^ Department of Biological Sciences, National Sun Yat-Sen University, Kaohsiung, Taiwan; ^9^ Department of Medical Research, Kaohsiung Medical University Hospital, Kaohsiung, Taiwan; ^10^ National Laboratory Animal Center, National Applied Research Laboratories, Taipei, Taiwan

**Keywords:** pancreatic ductal adenocarcinoma, tumour microenvironment, desmoplasia, immunomodulation, chemoresistance, ICI resistance

## Abstract

Pancreatic ductal adenocarcinoma (PDAC) is an aggressive and lethal cancer with a dismal five-year survival rate of 11%. Despite remarkable advancements in cancer therapeutics, PDAC patients rarely benefit from it due to insurmountable treatment resistance. Notably, PDAC is pathologically characterized by an extensive desmoplastic reaction and an extremely immunosuppressive tumour microenvironment (TME). The PDAC TME consists of cell components (e.g., tumour, immune and stromal cells) and noncellular components (e.g., extracellular matrix), exhibiting high complexity and their interplay resulting in resistance to chemotherapeutics and immune checkpoint inhibitors. In our review, we shed light on how crosstalk of complex environmental components modulates PDAC drug resistance, and we summarize related clinical trials. Moreover, we extend our discussion on TME exploration and exosome analysis, providing new insights into clinical applications, including personalized medicine, disease monitoring and drug carriers.

## Introduction

Pancreatic ductal adenocarcinoma (PDAC) is an aggressive and lethal cancer type with a dismal five-year survival rate of 11% (Siegel et al., 2022). According to National Comprehensive Cancer Network guidelines, surgical resection is the only potentially curative approach (Tempero et al., 2019). Unfortunately, it is estimated that approximately 80% of PDAC patients are diagnosed at an advanced or metastatic stage (Siegel et al., 2022). In other words, only 20% of PDAC patients are appropriate candidates for surgery (Moletta et al., 2019). Thus, systemic chemotherapy is the mainstay treatment for most patients (Sohal et al., 2016). Nevertheless, the clinical outcome is not significantly encouraging due to the occurrence of chemoresistance (Ducreux et al., 2019). The emergence of immune checkpoint inhibitors (ICIs) has revolutionized cancer treatment and brought benefits, especially for patients with haematological malignancies (Armand, 2015). However, PDAC patients rarely benefit from ICIs due to poor response (O'Reilly et al., 2019). Based on this fact, further investigation of drug mechanisms and the development of more effective regimens to overcome drug resistance are urgently needed for PDAC patients.

A large body of studies have indicated that PDAC is characterized by extensive desmoplasia. Its acellular matrix can constitute up to 90% of PDAC tumour bulk (Neesse et al., 2011; Torphy et al., 2018), and the tumour microenvironment (TME) consists of nonmalignant cells (e.g., stromal and immune cells) and noncellular components (e.g., collagen, glycoprotein and proteoglycans), indicating its complexity and desmoplasia (Feig et al., 2012; Tian et al., 2019). Notably, the PDAC TME is the culprit in insurmountable treatment resistance (Capurso and Sette, 2019), and it also contributes to recurrence and metastatic spread, which are also critical issues for PDAC patients (Steele et al., 2016; Brooks et al., 2018). Considering that radiotherapy is relatively less frequently prescribed than systemic chemotherapy due to late diagnosis of the disease (Orth et al., 2019; Siegel et al., 2022), and that a large number of genetic alterations are ranked as level II to IV in PDAC according to the ESMO Scale for Clinical Actionability of molecular Targets (ESCAT) (Mosele et al., 2020), we mainly focused on treatment resistance to chemotherapeutics and immunotherapy. Therefore, in this review, we shed light on the complex relationships between tumours and the TME, emphasizing how stromal-immune crosstalk exacerbates PDAC resistance to chemotherapy and ICIs.

## PDAC TME and chemoresistance

As mentioned previously, nearly 80% of PDAC cases are inoperable due to the time of diagnosis at an advanced or metastatic stage, urgently requiring systemic chemotherapy (Siegel et al., 2022). For locally advanced or metastatic PDAC patients with good performance status (PS), gemcitabine (GEM, also known as dFdC)/nanoalbumin-bound paclitaxel (Nab-PTX) or FOLFIRINOX (including leucovorin, 5-FU, irinotecan and oxaliplatin) are recognized as preferred first-line regimens to prolong survival (Tempero et al., 2021a). Conversely, GEM monotherapy is preferred for unresectable patients with poor PS due to less toxicity (Tempero et al., 2021a). Nevertheless, most patients develop chemoresistance, likely followed by local recurrence or metastatic spread. Given that GEM-based regimens are the standard of care for inoperable PDAC cases, we mainly shed light on GEM resistance in the following section.

### PDAC TME confers gemcitabine resistance through modulating drug actions

**FIGURE 1:**
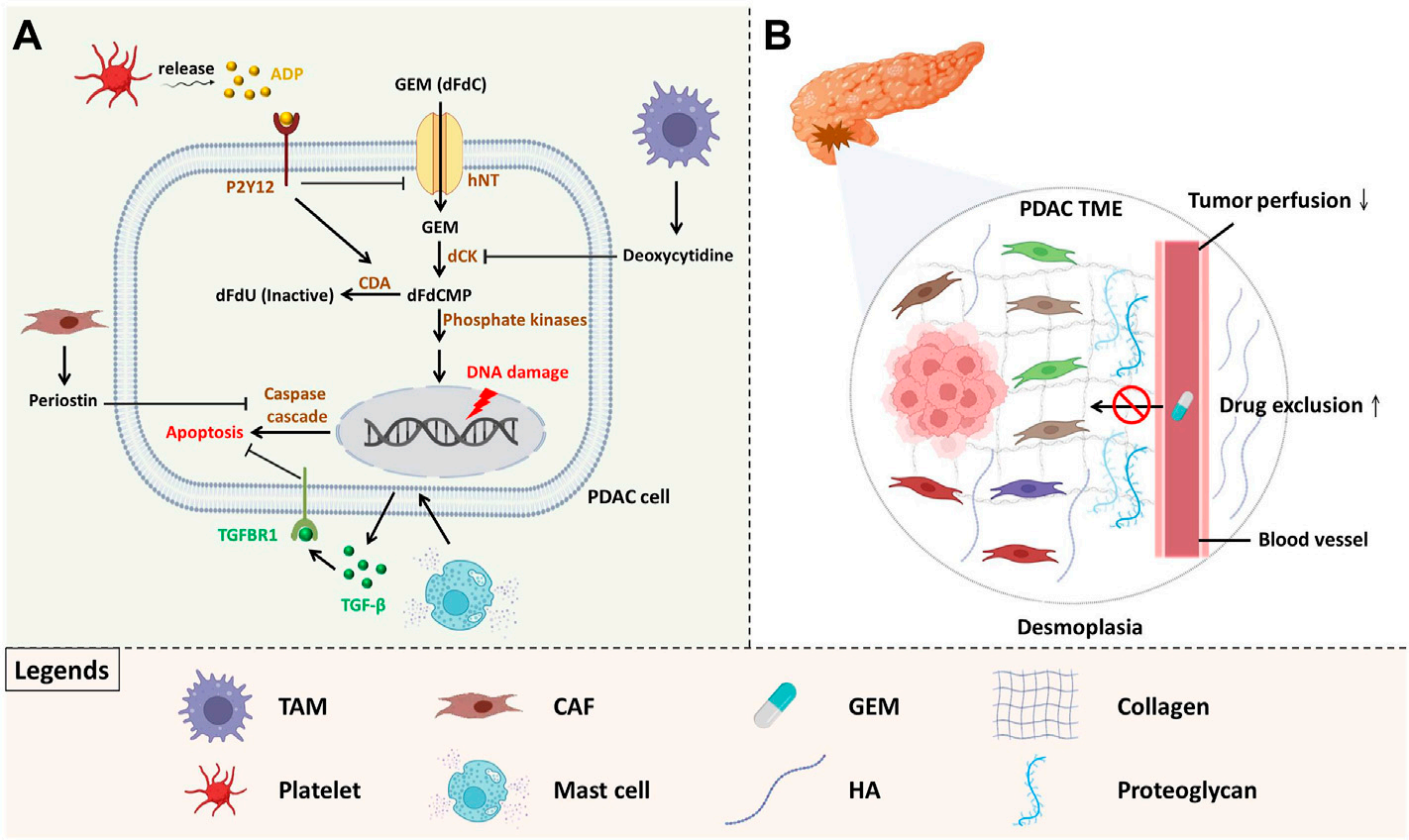
The components within the PDAC TME facilitate chemoresistance. **(A)** Chemoresistance conferred by tumour microenvironmental cells GEM resistance can be conferred by the components within the PDAC TME by modulating drug metabolism and enhancing antiapoptotic effects. **(B)** Desmoplasia and poor tumour perfusion spatially exclude GEM Activated CAFs induce ECM deposition, resulting in poor perfusion, forming a physical barrier and considerably compromising the access of GEM to tumour compartments.

Mechanistically, GEM uptake is mediated by human nucleoside transporter (hNT) and converted into gemcitabine monophosphate (dFdCMP) through deoxycytidine kinase (dCK), finally transforming into an active form to exert cytotoxicity by subsequent phosphate kinases. Conversely, cytidine deaminase (CDA) deactivates GEM to 2′,2′-difluorodeoxyuridine (dFdU), compromising its activity (Qin et al., 2020). Accumulating evidence has indicated that GEM resistance can be conferred by components within the PDAC TME through modulating drug metabolism (Figure 1A). Halbrook and colleagues discovered that tumour-associated macrophage (TAM)-derived pyrimidine metabolites, especially deoxycytidine, competitively interact with dCK, resulting in a reduced level of active GEM (Halbrook et al., 2019). Another study demonstrated that platelet-derived ADP binds with P2Y12 (ADP receptor) on PDAC, contributing to hNT downregulation and CDA upregulation (Elaskalani et al., 2017). GEM-elicited cytotoxicity is induced by DNA damage-associated apoptosis, and a previous study reported that deficient proapoptotic effects contributed to chemoresistance (Figure 1A) (Li et al., 2020a). Mast cells (MCs), which are primarily responsible for allergic reactions, are also involved in PDAC tumorigenesis (Krystel-Whittemore et al., 2015). Porcelli et al. revealed that MCs drive PDAC drug resistance through TGF-β signalling activation and upregulated antiapoptotic effects. However, administration of the TGF-β receptor type I inhibitor galunisertib restored GEM cytotoxicity. Notably, the serum level of MC tryptase increased in unresponsive patients, potently serving as an indicator of treatment response (Porcelli et al., 2019). Cancer-associated fibroblasts (CAFs), located in the stroma, are involved in several cancer processes, such as angiogenesis and immunosuppression (Li et al., 2020b; Francescone et al., 2021). Pancreatic stellate cells (PSCs), a well-investigated source of CAFs within the pancreatic stroma, are physiologically involved in extracellular matrix (ECM) homeostasis (Apte et al., 2012). However, ECM dysregulation contributes to PDAC progression. It is evident that periostin upregulation in PSCs enhances GEM resistance by blocking cleaved caspase-9 and DNA damage-triggered apoptosis (Liu et al., 2016).

### PDAC TME confers gemcitabine resistance through disrupting drug delivery

ECM protein deposition driven by activated CAFs forms a physical barrier that compromises drug entry and is associated with poor prognosis (Hingorani et al., 2018; Gorchs et al., 2019). Moreover, a unique pathological feature of PDAC is hypovascularity, which is mediated by high interstitial fluid pressure generated by excessive ECM deposition and closely correlated with reduced chemotherapeutic agent efficacy and poor prognosis (Figure 1B) (Di Maggio et al., 2016; Katsuta et al., 2019). Zhang et al. demonstrated that PDAC-secreted IL-1β stimulates CAFs and tumour fibrosis *via* IRAK4/NF-kB signalling, forming a shield to exclude GEM (Zhang et al., 2018). Heparan sulphate proteoglycan 2 (a multifunctional proteoglycan) derived from CAFs not only diminished the efficacy of GEM/Nab-PTX but also established a prometastatic niche *via* NF-kB paracrine signalling (Vennin et al., 2019). Overexpressed hyaluronic acid (HA) secreted by CAFs also compromises perfusion and drug entry. Notably, in a phase II clinical study (NCT01839487), improved PFS was observed in patients with stage IV PDAC after treatment with PEGPH20 (PEGylated recombinant human hyaluronidase) plus GEM/Nab-PTX (Hingorani et al., 2018). Rho-associated protein kinase 1 (ROCK1), which functions in controlling cell motility, is overexpressed in CAFs and is highly correlated with CAF activation, as well as ECM deposition (Whatcott et al., 2017). Vennin and colleagues found that inhibition of ROCK by the Rho kinase inhibitor fasudil stimulated ECM remodelling *in vivo*, not only compromising metastatic spread and liver colonization but also improving sensitivity to GEM/Nab-PTX (Vennin et al., 2017). Furthermore, Whatcott *et al.* reported that treatment with fasudil resulted in increased mean dFdCMP concentrations, indicating that ROCK inhibition attenuates CAF-induced ECM accumulation and drug exclusion (Whatcott et al., 2017). Recently, several studies have suggested that vitamin D receptor (VDR) signaling activation can drive CAFs into a quiescent state, concomitant with decreased ECM deposition and increased vascular lumen size. It was evident that intratumoural concentrations of dFdCTP, also an active form of GEM, increased significantly *in vivo* after treatment with the vitamin D analogue calcipotriol (Sherman et al., 2014). Another study conducted by Kim demonstrated that pretreatment with calcipotriol alleviated fibrosis, which not only improved chemotherapy delivery, but also promoted oncolytic virus-mediated antitumour immunity through increased immune cells recruitment and reduced T cell exhaustion. This indicated calcipotriol can serve as a promising adjuvant to enhance viroimmunotherapy efficacy by loosing PDAC dense stroma beforehand (Kim et al., 2022). Based on the rationale, several clinical trials are ongoing to assess the effectiveness of vitamin D ligands on reprogramming the PDAC TME. For instance, a phase I/II clinical study (NCT03520790) investigated the safety as well as efficacy of paricalcitol (a vitamin D analogue approved by the FDA to treat chronic renal failure-related hypercalcaemia) plus GEM/Nab-PTX in patients with metastatic PDAC. Another randomized clinical trial (NCT02030860) evaluated neoadjuvant paricalcitol with GEM/Nab-PTX in resectable PDAC patients. In addition to VDR signaling activation, photodynamic therapy (PDT) has also been reported to decrease ECM density, increase collagen nonalignment, and improve drug delivery (Obaid et al., 2019; Obaid et al., 2022). Patients who are refractory to GEM require high-dose administration, but intolerable toxicities are major concerns. Anbil and colleagues revealed that photodynamic priming coupled with calcipotriol can effectively increase intratumoural accumulation of chemotherapeutic agents by suppressing CXCL12/CXCR7 crosstalk and promoting vascular permeability. Hence, this strategy improved patients’ tolerability but maintained treatment efficacy (Anbil et al., 2020).

Intriguingly, metformin, widely prescribed for patients with type 2 diabetes mellitus, was suggested to reduce desmoplasia through inhibiting collagen I and hyaluronan production from CAFs by downregulating angiotensin-II receptor 1 (AT-1)/transforming growth factor-beta (TGF-β) signalling (Incio et al., 2015). Indeed, in a phase II clinical trial (NCT02005419), the activities of metformin combined with GEM were evaluated in resectable PDAC patients. Another phase II clinical study (NCT01210911) aimed to determine the activity of metformin with erlotinib and GEM in patients with locally advanced or metastatic PDAC.

Collectively, apart from the intrinsic resistance of PDAC cells, the components within the PDAC TME also facilitate chemoresistance. Importantly, the contributing factors primarily include resistance abilities conferred by surrounding cells and drug exclusion driven by dense stroma along with poor tumour perfusion.

## PDAC TME and ICI resistance

### PDAC TME confers ICI resistance through modulating immunogenicity

**FIGURE 2:**
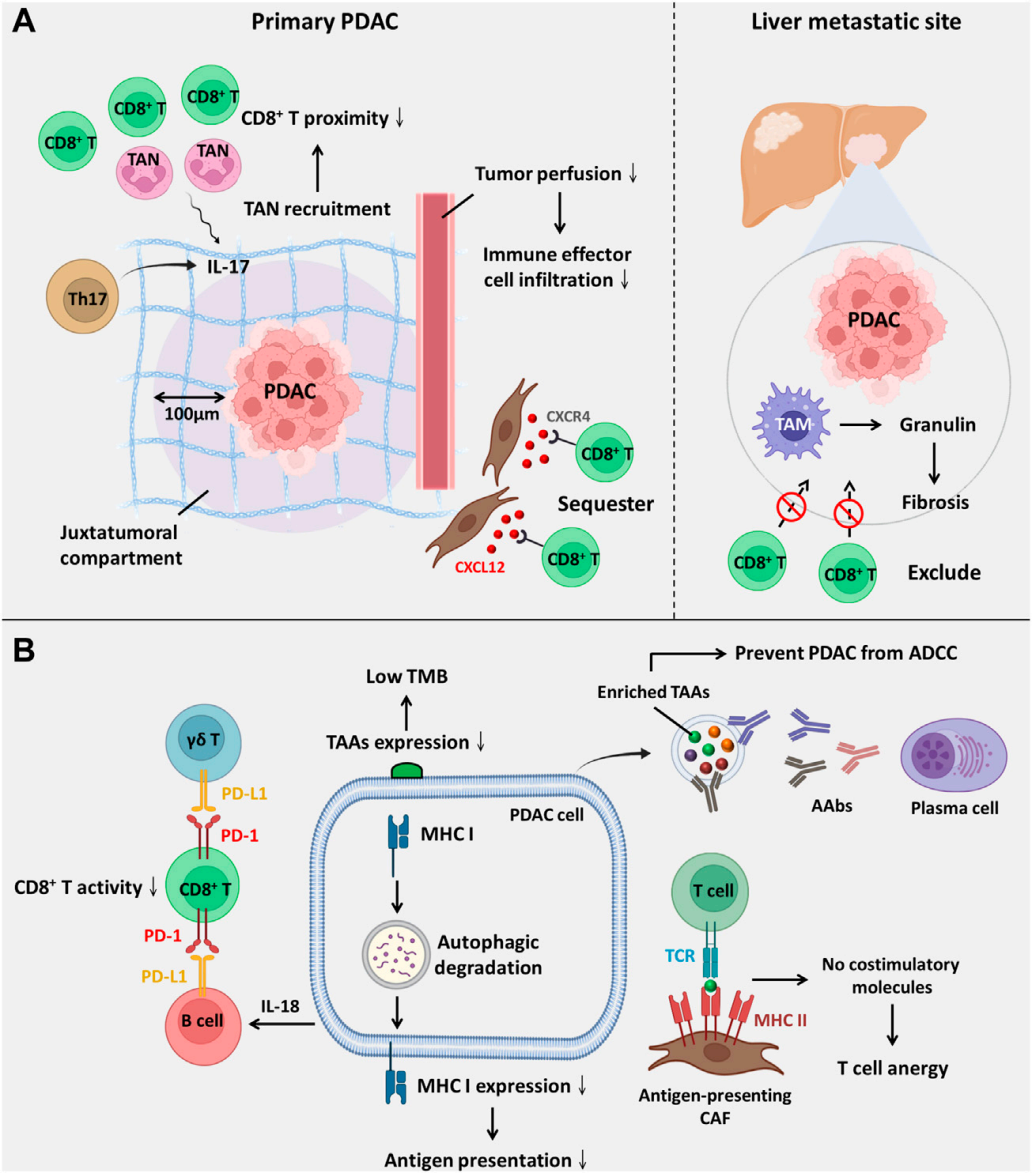
The components within the PDAC TME facilitate ICI resistance. **(A)** ICI resistance mediated by spatial exclusion of CD8^+^ T cells is primarily facilitated by the accumulation of tumour-infiltrating neutrophils and excessive ECM deposition. **(B)** ICI resistance induced by low immunogenicity and dysfunction of CD8^+^ T-cell ICI efficacy is compromised by downregulated antigenic presentation and reduced CD8^+^ T-cell activity. Abbreviations: AAbs, autoantibodies; ADCC, antibody-dependent cell-mediated cytotoxicity; CXCL12, C-X-C motif chemokine ligand 12; CXCR4, C-X-C motif chemokine receptor 4; TAAs, tumour-associated antigens; TAM, tumour-associated macrophage; TAN, tumour-associated neutrophil; TCR, T-cell receptor; TMB, tumour mutation burden.

ICIs have attracted much attention in recent years and have brought benefits to patients with haematological malignancies (Armand, 2015). Nevertheless, PDAC patients rarely benefit from ICIs owing to poor response (O'Reilly et al., 2019). The poor response to ICIs is primarily attributed to a low tumour mutation burden, an immunosuppressive TME and physical barriers (Yarchoan et al., 2017; Hester et al., 2021). Indeed, PDAC escapes immune surveillance since tumour-associated antigens (TAAs) are usually limited and absent (Fan et al., 2020). Autoantibodies (AAbs) are antibodies against TAAs. PDAC-derived extracellular vesicles are enriched in TAAs that serve as competitive binding sites for AAbs, which protect tumour cells from antibody-dependent cell-mediated cytotoxicity (Capello et al., 2019). Heterogeneous expression of MHC I occurs in PDAC because neighbour of BRCA1 gene 1 (NBR1), an autophagy receptor, interacts with MHC I to induce its autophagic degradation. Lysosomal inhibition through BafA1 and CQ upregulates MHC I and sensitizes PDAC to ICIs (Yamamoto et al., 2020). Furthermore, Elyada and colleagues found a CAF subpopulation called antigen-presenting CAFs that express high levels of MHC II without costimulatory molecules, such as CD80, CD40 and CD86, expressed by DCs. This situation leads to T-cell anergy and also promotes immunosuppressive CD4^+^ FOXP3^+^ regulatory T-cell (Treg) accumulation (Figure 2B) (Elyada et al., 2019).

### PDAC TME confers ICI resistance through affecting the spatial proximity of CD8^+^ T cells

It has been suggested that the spatial proximity of CD8^+^ T cells to tumours is highly associated with the ICI response (Figure 2A) (Slagter et al., 2020). Zhang and colleagues showed that T helper 17-cell-secreted IL-17 induced neutrophil recruitment, excluding CD8^+^ T cells from PDAC, and triggered ICI resistance. IL-17 neutralization alters spatial distribution and promotes CD8^+^ T-cell proximity to PDAC while upregulating PD-L1 mRNA expression. Hence, dual administration of an anti-IL-17 mAb and an ICI exerts a synergistic effect on PDAC regression (Zhang et al., 2020). Another study revealed that macrophage-derived granulin induces fibrosis to exclude CD8^+^ T-cell infiltration from the liver metastatic site instead of influencing their functions. This outcome likely explains ICI resistance in metastatic PDAC (Quaranta et al., 2018). Interestingly, one study showed that activated PSCs secrete C-X-C motif chemokine ligand 12 (CXCL12) to sequester CD8^+^ T cells, reducing CD8^+^ T-cell infiltration in the juxtatumoural compartment (identified as <100 μm from the tumour) (Ene-Obong et al., 2013). Administration of BL-8040, a C-X-C motif chemokine receptor 4 (CXCR4) antagonist, increases the density of CD8^+^ T cells and suppresses myeloid-derived suppressor cells (MDSCs) and Tregs within the intratumoural stroma. It was also found that BL-8040 outweighs other CXCR4 antagonists owing to its superior affinity. In a phase II clinical study, a cohort of metastatic PDAC patients with chemoresistance who received BL-8040 plus pembrolizumab (anti-PD-1 blocker) showed a 43.5% disease control rate and a 7.5-month median overall survival (Bockorny et al., 2020). Similar to chemoresistance driven by drug exclusion, the ICI response is also determined by the density of the stroma. Koikawa et al. emphasized that proline isomerase peptidyl-prolyl cis/trans isomerase NIMA-interacting 1 (PIN1) is overexpressed in CAFs and PDAC and that its overexpression also correlates with poor T-cell infiltration due to an active desmoplastic reaction. However, *in vivo*, inhibition of PIN1 by all-trans retinoic acid and arsenic trioxide or sulfopsin alleviates ECM deposition and improves anti-PD-1 blockade sensitivity (Koikawa et al., 2021). As mentioned previously, VDR signaling activation can reprogram PDAC stroma and reduce ECM deposition (Sherman et al., 2014). Of note, it was evident that autophagy contributed to VDR signaling inactivation within PSCs (Kong et al., 2022). Based on this fact, Kong and associates utilized pH-buffering micelles encapsulated with calcipotriol to block autophagic flux and synergistically reprogram PSCs *in vitro* and *in vivo*, which enhanced the PDAC response to anti-PD-1 blockade (Kong et al., 2022). A phase II clinical study (NCT03331562) evaluated whether the effect of pembrolizumab (anti-PD-1 blockade) could be enhanced by addition of calcipotriol in PDAC patients. However, recent evidence in 2D or 3D cell culture system reveals although calcipotriol can inhibit CAFs proliferation as well as migration, it unexpectedly upregulates PD-L1 and suppresses T cell activity (Gorchs et al., 2020). Hence, further clinical investigation is urgently needed. Intriguingly, CAFs are not always involved in establishing dense stroma; conversely, they can also facilitate PDAC progression by alleviating desmoplastic reactions. Wang and associates identified a novel subset of CAFs with high expression of phospholipase A2 group IIA, called metabolic state CAFs (meCAFs), by single-cell RNA sequencing. These cells predominate in loose-type PDAC, whereby a loose stroma facilitates CD8^+^ T-cell infiltration and cytotoxicity but promotes metastasis. Hence, an abundance of meCAFs is linked to poor prognosis, although it indicates a better response to immunotherapy; it is estimated that PDAC patients with abundant meCAFs have a 64.7% response rate to PD-1 inhibitors (Wang et al., 2021a).

### PDAC TME confers ICI resistance through affecting the function of CD8^+^ T cells

Apart from the spatial distribution of CD8^+^ T cells, the ICI response is also influenced by its activity and function (Figure 2B), which are primarily mediated by interactions between inhibitory checkpoint molecules and their corresponding inhibitory receptors. Lymphocyte-activation gene 3 (LAG-3), a coinhibitory molecule, impairs CD8^+^ T-cell function. Additionally, LAG-3^+^ T-cell infiltration is related to reduced DFS (Long et al., 2018); in contrast, a high inducible costimulator positive (ICOS^+^) T-cell density is suggestive of extended DFS since ICOS is a paramount stimulatory checkpoint molecule for T-cell activation (Seifert et al., 2021). Disappointingly, ICOS expression on Tregs is higher than that on CD8^+^ T cells. Nevertheless, KY-1044, a selective anti-ICOS blocker, preferentially inhibits tumour-infiltrating ICOS-high Tregs. The safety, tolerability and efficacy of KY-1044 with atezolizumab were evaluated in advanced PDAC in a phase I/II clinical trial (NCT03829501) (Quaratino et al., 2019). γδ T cells are minor T-cell lineages and non-MHC-restricted lymphocyte subsets involved in innate immunity (Daley et al., 2020). However, a recent study uncovered their role in PDAC tumorigenesis. Daley *et al.* revealed that tumour-infiltrating γδ T cells induce dysfunction of CD8^+^ T cells *via* upregulation of inhibitory ligands (e.g., PD-L1 and galectin-9 (Gal-9)). High expression of Gal-9 was observed in γδ T cells, inducing M2 polarization and inhibiting T cells. Notably, serum Gal-9 is able to distinguish PDAC cases from benign or healthy subjects (HS) (Seifert et al., 2020). Surprisingly, γδ T cells constitute up to 75% of all T lymphocytes in the human PDAC stroma, and depletion of γδ T cells not only leads to extended survival but also induces T helper 1 (Th1) cell differentiation and CD8^+^ T-cell cytotoxicity (Daley et al., 2020). Over the past few decades, the role of B cells in solid tumours has been neglected and underrated. Bruton tyrosine kinase (BTK) is an important kinase for promoting B-cell activity and M2 polarization. Administration of the FDA-approved BTK inhibitor ibrutinib restores CD8^+^ T-cell cytotoxicity (Gunderson et al., 2016). Interestingly, emerging evidence has indicated that B cells are also one of the sources of PD-L1. Activated B cells suppress CD8^+^ T-cell activity and IFN-γ generation in a PD-L1-dependent manner. Additionally, elevated PD-L1 expression on circulating B cells was observed in patients with advanced PDAC rather than HS (Tong et al., 2020). Zhao *et al.* reported that PDAC-producing IL-18 promotes IL-10 secretion by regulatory B cells and induces PD-L1 expression on B cells to inhibit cytotoxic T cells with reduced granzyme B and IFN-γ (Zhao et al., 2018).

A previous study indicated that increasing resistance of prostate cancer to ipilimumab (anti-CTLA-4 mAb) could be attributed to a compensatory inhibitory pathway. In other words, CTLA-4 blockade induces the upregulation of other inhibitory checkpoint molecules, including PD-1, PD-L1 and V-domain immunoglobulin suppressor of T-cell activation (VISTA) (Gao et al., 2017). A similar phenomenon was also observed in PDAC. Hou et al. indicated that VISTA is highly expressed on PDAC and M2 macrophages in tumour tissues, causing CD8^+^ T-cell exhaustion (Hou et al., 2021). The safety and recommended effective dose of JNJ-61610588 (anti-VISTA mAb) in PDAC patients were evaluated in a phase I clinical study (NCT02671955). Unfortunately, the study was terminated owing to the manufacturer’s decision. Nonetheless, VISTA remains a promising target for patients with pancreatic cancer (Blando et al., 2019).

To conclude, the poor response to ICIs in PDAC is primarily attributed to low immunogenicity, spatial exclusion of CD8^+^ T cells by a strong desmoplastic reaction and suppressed activity of cytotoxic T cells, facilitated by immunosuppressive cell trafficking to the TME.

## Clinical trials: Strategies that modulate PDAC TME to restore drug sensitivity

PDAC is characterized by an extensive desmoplastic reaction and immunosuppressive TME, making substantial contributions to restraining clinically prescribed chemotherapy and compromising ICI efficacy. In recent years, a growing number of studies have demonstrated that modulating the PDAC TME not only boosts the efficacy of chemotherapy and ICIs but also leads to tumour regression, along with an improved survival rate in *in vivo* preclinical models. Among these strategies, targeting stromal desmoplasia (e.g., alleviating ECM deposition) and restoring tumour immunosurveillance (e.g., promoting CD8^+^ T-cell infiltration and function) have attracted much attention from researchers. Since standard-of-care regimens remain important for tumour elimination, most clinical trials are designed to evaluate the efficacy of combinations of TME-modulated agents with standard-of-care drugs. The following table (Table 1) briefly summarizes and describes the related clinical trials. Most clinical studies are in phase I or II to evaluate drug tolerability, safety and side effects; several studies have reported promising outcomes. For instance, in a phase II study (NCT01373164), galunisertib (an inhibitor of TGF-β receptor I) plus GEM showed improved OS with manageable toxicities compared to GEM plus placebo (Melisi et al., 2018). Another phase II study (NCT01839487) reported that patients with HA-high tumours largely benefited from PEGPH20 (recombinant human hyaluronidase) in PFS (Hingorani et al., 2018). Nevertheless, some trials were terminated due to lack of funding, slow accrual and unexpected adverse events. Unfortunately, in a phase III study (NCT02436668), ibrutinib plus GEM/nab-PTX showed no improvement in OS and even shorter PFS in metastatic PDAC (Tempero et al., 2021b). Despite this setback, the continued development of TME modulation is ongoing and promising in improving therapeutic efficacy, but it requires further investigation.

**TABLE 1:** Clinical trials of strategies for conditioning the PDAC TME.

Phase	NCT number	Status	Condition/disease	Intervention/treatment	Description
Not applicable	NCT02030860	Completed	Resectable PDAC	Neoadjuvant paricalcitol with GEM/Nab-PTX	To evaluate the effect of paricalcitol plus GEM/Nab-PTX
I	NCT01777477	Completed	Unresectable and metastatic PDAC	Chloroquine plus GEM	To evaluate combination safety, MTD and efficacy
I/II	NCT03520790	Recruiting	Metastatic PDAC	Paricalcitol plus GEM/Nab-PTX	To evaluate the efficacy
I/II	NCT01373164	Completed	Unresectable PDAC	Galunisertib with GEM	To evaluate the safety, tolerable dose and OS
I/II	NCT02403271	Completed	Relapsed or refractory PDAC	Ibrutinib with durvalumab	To evaluate the safety, tolerability, and efficacy
I/II	NCT03829501	Recruiting	Advanced PDAC	KY-1044 with atezolizumab	To evaluate safety, tolerability, and efficacy
II	NCT02005419	Completed	PDAC after curative resection	GEM plus metformin	To evaluate combination activity and safety
II	NCT01210911	Completed	Locally advanced or metastatic pancreatic cancer	GEM, metformin and erlotinib	To determine the activity and safety
II	NCT01839487	Completed	Metastatic PDAC	PEGPH20 and GEM/nab-PTX (PAG)	Improved PFS was observed in PAG treatment, and the longest PFS was in patients with HA-high PDAC receiving PAG.
II	NCT03331562	Completed	Pancreatic cancer	Pembrolizumab plus paricalcitol	To evaluate the combination effect for the maintenance of pancreatic cancer
II	NCT02907099	Active, not recruiting	Recurrent or metastatic PDAC	BL-8040 with pembrolizumab	To assess ORR and T-cell infiltration
II	NCT03634332	Unknown	HA-high metastatic PDAC	PEGPH20 and pembrolizumab	To evaluate the efficacy of combination
III	NCT02436668	Completed	Metastatic PDAC	Ibrutinib and GEM/nab-PTX	To assess safety, OS, and PFS.

HA, hyaluronic acid; MTD, maximum tolerated dose; OS, overall survival; ORR, overall response rate; PFS, progression-free survival.

## Conclusion

Mounting evidence has indicated that the dismal prognosis of PDAC can be primarily attributed to treatment resistance and the limitations of early detection. PDAC is characterized by an extensive desmoplastic reaction and an extremely immunosuppressive TME, also known as a “cold tumour”. The complexity of the PDAC TME makes substantial contributions to restraining therapeutic efficacy, particularly with chemotherapy and immunotherapy, and so that subsequent local recurrence or metastatic relapse likely occurs. Regarding chemoresistance, the contributing factors include (Siegel et al., 2022): dense stroma; along with hypovascularity to exclude chemodrugs; and (Tempero et al., 2019) resistance abilities conferred by the components of the TME through altering of drug metabolism or upregulating of anti-apoptotic effects. The poor response to ICI is mainly due to (Siegel et al., 2022): low TMB (Tempero et al., 2019); establishment of a physical barrier to spatially preclude cytotoxic T cells; and (Moletta et al., 2019) dysfunction of cytotoxic T cells mediated by immunosuppressive cells. In conclusion, a comprehensive investigation of the characteristics of the PDAC TME might facilitate the design of feasible combination regimens to improve therapeutic efficacy and prolong patient survival.

## Discussion

### Predicting drug efficacy by exploring components of PDAC TME

**FIGURE 3:**
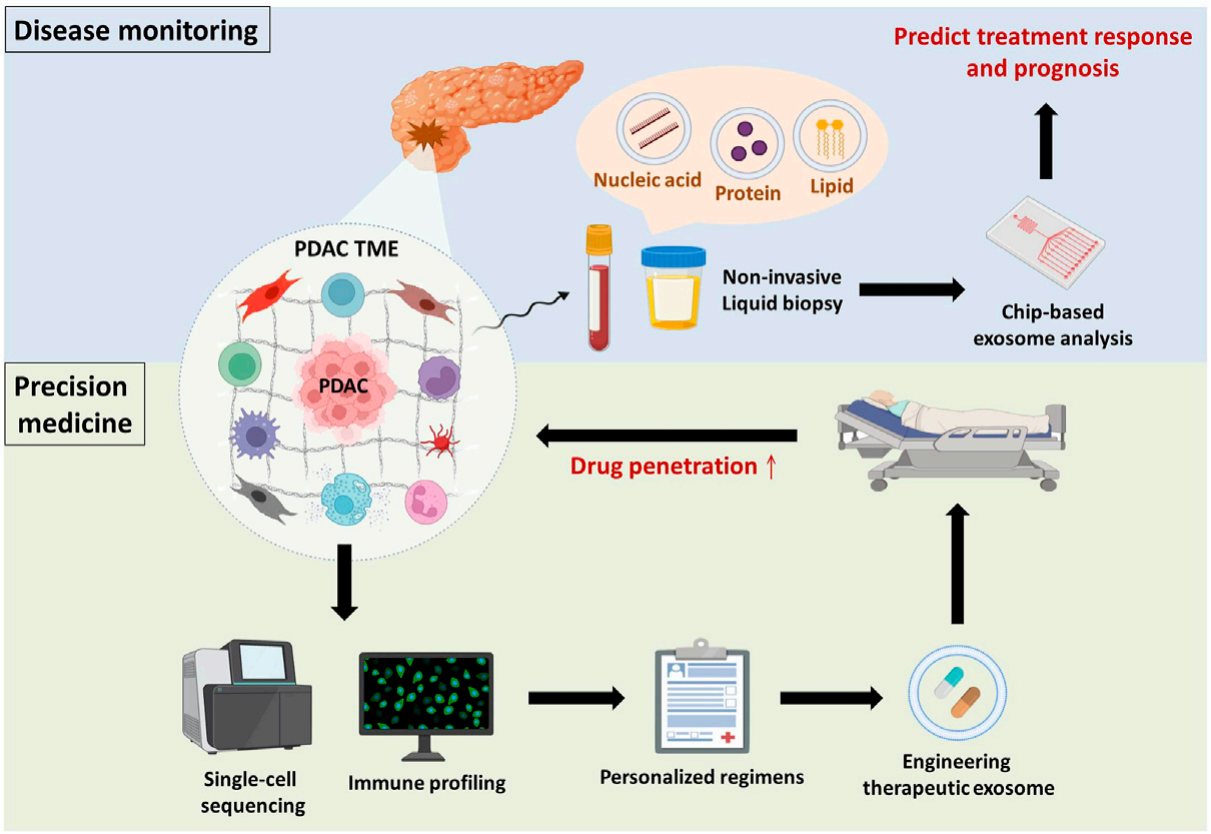
Overview and future perspectives. Disease monitoring: PDAC or tumour microenvironmental cell-derived exosomes are detected in body fluids (e.g., blood and urine), and their cargos are indicative of PDAC progression. Taking advantage of chip-based exosome analysis can immediately predict treatment response and prognosis. Precision medicine: Components of the TME (e.g., immune cells, stromal cells and ECM) make substantial contributions to PDAC progression. Hence, single-cell sequencing of tumour microenvironmental components in combination with immune profiling provides physicians with comprehensive information to establish personalized regimens. Subsequently, drug candidates can be encapsulated in exosomes to improve drug penetration.

Over the past few decades, cancer treatment has mainly depended on the histological features of cancer cells and AJCC/UICC TNM staging. With remarkable advances in gene sequencing, oncologists have focused attention on precision medicine, and specific gene mutations are now considered biomarkers or therapeutic targets (e.g., *EGFR* mutations in non-small-cell lung cancer and Gefitinib) (Nan et al., 2017). Recently, immune profiling has developed and attracted much attention. Immune profiling refers to deep analysis of immune cell phenotypes within the TME. Parra and colleagues established and optimized a novel immune-profiling methodology, namely the automated multiplex immunofluorescence panel, to examine the density and spatial distribution of specific immune cells within the stromal or tumour compartments (Parra et al., 2021). Chauh *et al.* reported that high-dimensional technologies, such as single-cell RNA-Seq (scRNA-Seq), cytometry by time of flight and multiplex immunohistochemistry, are accessible for evaluating rare immune cell subsets and overcoming heterogeneity, profiling the immune context of the TME to accurately predict prognosis and immunotherapy-related adverse effects (Chuah and Chew, 2020). Additionally, Lenzo *et al.* conducted immune profiling of PDAC patients by quantifying cell types using scRNA-seq and evaluating the spatial distribution of CD8^+^ T cells with immunohistochemistry (Lenzo et al., 2021). Garner claimed that binary classification of immune cells, such as TAMs and tumour-associated neutrophils (TANs), is insufficient for the accurate assessment of phenotypes. According to single-cell sequencing, TAMs can coexpress M1-and M2-related genes, and TANs could exhibit a spectrum of phenotypes. Furthermore, single-cell transcriptomics identified 25 distinct tumour-infiltrating myeloid cell states. Such findings raise concerns about whether single-cell sequencing can actually model the sophistication of the TME (Garner and de Visser, 2020). The same phenomenon has been observed in CAFs. Stromal contents serve as “Jekyll and Hyde” in PDAC progression, which might be explained by the high heterogeneity and diverse subsets of CAFs. As mentioned previously, CAF-induced dense stroma forms a physical barrier to reduce drug efficacy and hypovascularity while simultaneously restraining PDAC cell invasion and metastasis (Wang et al., 2021a). Moreover, Tian and associates proteomically analysed PDAC ECM composition by liquid chromatography-tandem mass spectrometry and discovered that a stroma-derived ECM correlates with either better or poorer clinical outcomes. Intriguingly, changed expression of specific ECM types is capable of distinguishing PDAC from the premalignant stage or pancreatitis (Tian et al., 2019). Collectively, analysing subpopulations of CAFs and ECM composition within the TME should be given prominence, which would provide the opportunity to evaluate drug efficacy before administration and facilitate the design of precision medicine (Figure 3). However, the types of CAFs that are involved in the PDAC TME and the categories of ECM that should be recognized as favourable or unfavourable indicators require further investigation.

### Exosomes derived from PDAC TME as promising biomarkers

Accumulating studies have reported that small extracellular vesicles (e.g., exosomes) and their cargos (e.g., proteins and nucleic acids) are involved in modulating PDAC progression, including chemoresistance, through crosstalk between PDAC cells and immunosuppressive cells (Binenbaum et al., 2018; Xavier et al., 2021). Because exosomes are secreted by parental cells to deliver intracellular signals and are detectable in several body fluids, they are considered promising biomarkers for disease monitoring (Zhou et al., 2020a). Shao and colleagues developed microfluidic-based chips to detect the mRNA levels of exosomal cargos, potentially providing physicians with real-time information about patients’ response to chemotherapy (Shao et al., 2015). Indeed, with significant progress in microfluidics research, the use of chip-based assays for exosome isolation and analysis has gained popularity based on its advantages over conventional exosome isolation (i.e., ultracentrifugation), such as greater sensitivity, lower sample volume requirements, more rapid processing, lower reagent consumption, and greater cost effectiveness (Wang et al., 2021b). Furthermore, Melo *et al.* revealed that glypican-1 is upregulated in PDAC and was specifically detected on PDAC-derived exosomes but not on nontumourigenic cell-derived exosomes (Melo et al., 2015). Hence, there is potential that chip-based analysis of PDAC-derived exosomal cargos might be used in clinical applications to immediately evaluate PDAC patient response to GEM (Figure 3). Nevertheless, there are several urgent issues that require further investigation. Cancer is notorious for its high heterogeneity; hence, the identification of specific surface markers to distinguish the originating cells of exosomes is paramount to avoid false-negative results. In addition, the development of good manufacturing practices to standardize operating procedures and parameters across different platforms and optimize exosome processing should be considered.

### Exosomes as drug carriers to improve drug penetration

Apart from disease monitoring, drug accumulation in malignant lesions seems to be a critical problem that must be surmounted in PDAC because of the impenetrable dense stroma and poor perfusion. Exosomes have offered new insights into PDAC treatment for two main reasons (Siegel et al., 2022): exosomes easily overcome impenetrable barriers, such as the blood–brain barrier and dense stroma, in PDAC (Zhou et al., 2020b; Zhou et al., 2021); and (Tempero et al., 2019) exosomes have low immunogenicity compared to other nanoparticle vesicles because they are natural endogenous nanovesicles that avoid systemic allergic reactions (Samanta et al., 2018). Zhou and associates demonstrated that incorporating PTX and dFdCMP, an intermediate of GEM, into purified exosomes collected from bone marrow-derived mesenchymal stem cell supernatant led to superior penetrating ability and antitumor effects *in vitro* and *in vivo*. Encouragingly, only mild systemic toxicity was observed after intravenous injection of exosomes (Zhou et al., 2020b). Similar to taking advantage of exosomes as liquid biopsies, standardization of engineering therapeutic exosomes and their pharmacokinetics in the real world requires further investigation from the bench to the clinic (Figure 3).
